# Tamoxifen may prevent both ER+ and ER- breast cancers and select for ER- carcinogenesis: an alternative hypothesis

**DOI:** 10.1186/bcr1342

**Published:** 2005-11-21

**Authors:** Laura J Esserman, Elissa M Ozanne, Mitch Dowsett, Joyce M Slingerland

**Affiliations:** 1 Deptartment of Surgery, University of California San Francisco Medical Center, San Francisco, CA, USA; 2 Institute for Technology Assessment, Massachusetts General Hospital, Harvard Medical School, Boston, MA, USA; 3 Royal Marsden Hospital, London SW3 6JJ, UK; 4 Division of Hematology Oncology and Braman Breast Cancer Institute, University of Miami Sylvester Comprehensive Cancer Center, Miami, FL, USA

## Abstract

**Introduction:**

Breast Cancer Prevention Trial (BCPT) and Multiple Outcomes of Raloxifene (MORE) data have been interpreted to indicate that tamoxifen reduces the risk of ER+ but not ER- breast carcinogenesis. We explored whether these data also support an alternative hypothesis, that tamoxifen influences the natural history of both ER+ and ER- cancers, that it may be equally effective in abrogating or delaying ER- and ER+ carcinogenesis, and place selection pressure, in some cases, for the outgrowth of ER- cancers.

**Methods:**

BCPT and MORE data were used to investigate whether: first, tamoxifen could reduce equally the emergence of ER- and ER+ tumors; and second, tamoxifen could select a fraction of emerging ER+ cancers and promote their transformation to ER- cancers. Assuming that some proportion, Z, of ER+ tumors becomes ER- after tamoxifen exposure and that the risk reduction for both ER- and ER+ tumors is equal, we solved for both the transformation rate and the risk reduction rate.

**Results:**

If tamoxifen equally reduces the incidence of ER+ and ER- tumors by 60%, the BCPT results are achieved with a transformation of approximately Z = 20% of ER+ to ER- tumors. Validation with MORE data using an equal risk reduction of 60% associated with tamoxifen produces an almost identical transformation rate Z of 23%.

**Conclusion:**

Data support an alternative hypothesis that tamoxifen may promote ER- carcinogenesis from a precursor lesion that would otherwise have developed as ER+ without tamoxifen selection.

## Introduction

The prevailing wisdom is that tamoxifen and other antiestrogens are active in preventing the development or progression of estrogen receptor positive (ER+) breast cancers only. Efficacy of tamoxifen for ER+ breast cancer has been clearly demonstrated in both metastatic and adjuvant settings. The benefit from adjuvant tamoxifen therapy was restricted to ER+ breast cancers in the Early Breast Cancer Trialists' meta-analysis [1]. Tamoxifen does not inhibit proliferation of estrogen receptor negative (ER-) breast cancer cell lines in tissue culture or in murine xenograft models [1,2]. Recently, Allred *et al*. [3] tested a subset of patients from the National Surgical Adjuvant Breast and Bowel Project (NSABP) B-24 trial and demonstrated that the effectiveness of tamoxifen following lumpectomy and radiotherapy for ductal carcinoma *in situ *was limited to ER+ *in situ *cancers. Thus, it was not at all unexpected that results from the NSABP P-01 Breast Cancer Prevention Trial (BCPT), the study of tamoxifen use versus placebo, fit the paradigm that tamoxifen prevents ER+ tumors. Women in the tamoxifen arm of the BCPT trial developed ER+ tumors at an annual incidence that was 69% less than that of the placebo arm (5.02/1000 annually in placebo arm versus 1.58/1000 annually in tamoxifen arm). However, the incidence of ER- tumor development was not significantly different between the tamoxifen and placebo arms [4].

Evidence that tamoxifen efficacy in both the adjuvant and metastatic settings is limited to ER+ tumors does not exclude the possibility that, in the prevention setting, this drug could reduce both ER+ and ER- tumorigenesis and mediate concomitantly a selection pressure for emergence of ER- tumors in susceptible neoplastic cell subpopulations. In the NSABP B-18, B-22 and B-25 studies, women under 50 years old were not offered tamoxifen and all women over 50 years old received tamoxifen. Long term follow up of these studies suggests that tamoxifen exposure resulted in a higher likelihood that subsequent contralateral breast cancer would be ER- when compared to women who did not receive tamoxifen. The conventional interpretation of these data implies that the increased incidence of ER- tumors is a result of chemoprevention of ER+ tumors by tamoxifen. It is possible, however, that the observed data can be explained through another mechanism.

The distribution of the ER+ or ER- second primary cancers conditional on the estrogen receptor (ER) status of the first breast primary from long term follow-up of the 5,513 patients from NSABP studies B-18, B-22, and B25 is shown in Table 1. When these studies were initiated, it was standard to treat all postmenopausal women with hormone therapy, regardless of ER status, but tamoxifen was not thought to be effective in premenopausal women. Therefore, in the NSABP trials B-18, B-22, and B25, all postmenopausal women were treated with tamoxifen, but none of the premenopausal women (at baseline) were, allowing the opportunity to examine whether tamoxifen might affect the ER status of tumors that developed subsequently. Interestingly, of the women aged >50 years with ER+ primary breast cancer who received adjuvant tamoxifen and went on to develop a contralateral second primary, 44% of the cancers that developed were ER-. This is substantially higher than the proportion of ER- second primaries in the group of women aged <50 years who did not receive adjuvant tamoxifen: only 11% of second primaries were ER- when tamoxifen was not used [5]. Although the number of second primaries arising in this very large initial population was small, these data raise the possibility that tamoxifen exposure could potentially select for subsequent development of ER- cancers. These data challenged us to explore an alternative hypothesis to explain the prevention trial data. Data from the BCPT and the Multiple Outcomes of Raloxifene (MORE) studies were used to examine whether tamoxifen inhibits equally the emergence of ER- and ER+ tumors, but also promotes the transformation of a small proportion of premalignant or neoplastic ER+ lesions to become ER- tumors [5].

## Methods

The data from the BCPT and MORE prevention trials are shown in Table 2. In 1992, the NSABP began the P-01 BCPT to determine if tamoxifen, an antagonist to estrogen in breast tissue, was effective for breast cancer prevention. The study included 13,338 high-risk postmenopausal women who had a 5 year Gail risk of 1.67% or higher and were aged 35 years or older. Half were randomized to tamoxifen (6,681 women), and the rest to placebo (6,707 women) and followed for a median of 3.5 years [4]. The MORE study began in 1994 and explored the effect of raloxifene on bone density in postmenopausal women as a primary endpoint and the incidence of breast cancer as a secondary endpoint [6]. A total of 7,290 postmenopausal women aged 80 years or younger with osteoporosis were randomized to either raloxifene or placebo. The mean age of study participants was 66 years. The number of ER- and ER+ breast cancers that developed during each trial is given in Table 2 for both the placebo and chemoprevention arms.

**Figure 1:**
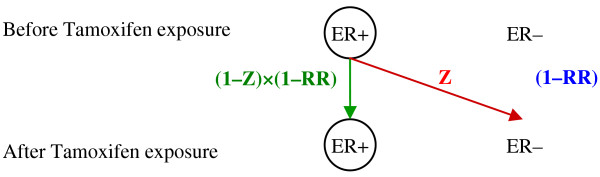
Equations used to test our alternative hypothesis. Rate of ER- tumor development equals Z(ER+) + (ER-) × (1 - RR). Rate of ER+ tumor development equals (1 - Z) × (ER+) × (1 - RR). ER+, percentage of ER- tumors in absence of tamoxifen; ER-, percentage of ER+ tumors in absence of tamoxifen; RR, risk reduction from tamoxifen; Z, rate of transformation from ER+ to ER- tumors.

A mathematical model was constructed to support the hypothesis that tamoxifen inhibits equally the development of ER- and ER+ tumors, and also promotes the transformation of a proportion of premalignant or neoplastic ER+ lesions to become ER- tumors. The model was developed under these assumptions, with unknown rates of transformation and risk reduction and was calibrated to match the observed data in these two chemoprevention trials, the BCPT and the MORE. The model's initial assumption was that tamoxifen reduced equally the development of both ER- and ER+ tumors. Second, the model assumed that some proportion, Z, of tumors that would otherwise become ER+ in the absence of tamoxifen would become ER- after exposure to tamoxifen. The tumors that transform from ER+ to ER- would be *de novo *tamoxifen resistant. The equations governing the model are shown in Fig. 1. The reference case assumes a 60% risk reduction for both ER- and ER+ tumors, based on oophorectomy data that show a 60% reduction in the risk of invasive breast cancer development [7]. Using this value for the risk reduction, the model can be used to solve for Z, the transformation rate to ER- tumors, fitting the data from the NSABP and MORE trials.

## Results

We assumed that if tamoxifen was equally effective in reducing ER- and ER+ cancers, the risk reduction might approximate that seen following oophorectomy in women with inherited mutations in *BRCA1/2*. Based on data that oophorectomy before the age of 40 years reduces the risk of invasive breast cancer development by approximately 60% [7], and solving for the number of ER- and ER+ tumors in the BCPT that emerged with and without therapy (Table 2), the transformation rate, Z, required to fit the BCPT data is equal to 20.1%. A very similar transformation rate of 22.7% is required to fit the MORE data when one sets the risk reduction to 60% for all breast cancers following tamoxifen use. Table 3 shows how the rate of transformation from ER+ to ER- tumors varies with different assumptions concerning the risk reduction from tamoxifen use. The values for Z are surprisingly similar between the BCPT and MORE data. Thus, we have demonstrated that the proportion of ER- and ER+ tumors can be explained if we assume that tamoxifen exposure results in an equal reduction in ER+ and ER- cancers but is also accompanied by the transformation to ER- of a small fraction of tumors that would otherwise have remained as ER+.

## Discussion

Mathematically, the BCPT and MORE data support the intriguing possibility that tamoxifen could exert an equal 60% risk reduction on ER- and ER+ tumor development, if there was an obligate transformation of approximately 20% of emerging tumors from ER+ to ER- during tumorigenesis. It is noteworthy that Kuukasjarvi and colleagues [8] have observed that approximately 20% of ER+ primary tumors can recur with ER- metastases even in the absence of medical therapy [8]. Similar transformation rates of ER+ to ER- at relapse during adjuvant tamoxifen therapy have also been reported [9,10].

There is also evidence that ER- cancers can emerge over time after exposure to tamoxifen. ER+ primary tumors may recur as ER- metastases in up to 20% of cases [8-10]. A recent meta-analysis of patients with ER+ or progesterone receptor positive (PR+) primary tumors showed 22% (range 0.17 to 0.3) of ER+ and 20% (range 0.2 to 0.33) of PR+ tumors recurred with hormone receptor negative disease [11]. Interestingly, prophylactic oophorectomy in *BRCA1 *and *BRCA2 *mutation carriers under the age of 40 years causes a 60% reduction in the risk of developing breast cancer. This is remarkable because 80% of *BRCA1 *tumors are ER-. These observations support the possibility that hormonal manipulations are effective in preventing the emergence of both ER+ and ER- tumors [7,12]. King *et al*. [13], in their subset analysis of cases from the BCPT, suggest that tamoxifen is effective in preventing breast cancer in women with *BRCA2 *mutations, but not *BRCA1 *mutations. Because the majority of *BRCA1 *tumors are ER-, these data question our assumption that tamoxifen does not prevent the outgrowth of ER- cancers. The number of mutation carriers in the BCPT study was small enough, however, that it is difficult to draw conclusions about these data in regards to our hypothesis; only 19 women were known to be *BRCA1 *or *BRCA2 *mutation carriers in the BCPT (8 *BRCA1 *and 11 *BRCA2*). However, we do know that ovarian suppression (a different form of estrogen suppression) does reduce the incidence of breast cancers in *BRCA1 *carriers. Furthermore, new data from Dr Narod's consortium suggest that tamoxifen does indeed prevent the development of breast cancers in *BRCA1 *carriers whose tumors are primarily negative (manuscript submitted; J. McClenann, personal communication).

Atypia, which is reported to be uniformly ER+ [14], can precede ER- cancers. In the BCPT, 1,193 women with atypia were entered into the study [4]. In the placebo and tamoxifen arms, 23 and 3 cancers developed, respectively. Of the cancers that developed, 6/23 (26%) compared to 1/3 (33%) were ER- in the placebo and tamoxifen treated arm, respectively. These numbers are too small to draw conclusions, but they are not inconsistent with the theory that ER- cancers can emerge from a previously ER+ preneoplastic cell population (J Costantino, personal communication) [4]. Taken together, these data suggest that exposure to tamoxifen could influence the development of a small fraction of ER- tumors, and led us to consider a reinterpretation of the prevention trial data.

Reduced levels of ER have been observed within 2 weeks of starting neoadjuvant therapy with tamoxifen or raloxifene [15,16], although the mechanism underlying this reduced expression is unclear. *In vitro*, the binding of oestradiol to ERs activates the transcriptional activity mediated by these receptors and also leads to enhanced proteasomal degradation of them [17-19]. It is possible that tamoxifen and raloxifene also elicit ER proteolysis. These early changes in ER levels may persist long-term and could lead to emergence of clinically apparent ER- tumors from subclinical ER+ tumors during tamoxifen or raloxifene therapy. These data and our modeling raise the possibility that tamoxifen could inhibit proliferation of both ER+ and ER- breast cancer cells but exert clonal selection for more proliferatively aggressive ER- clones during long-term tamoxifen therapy.

Several molecular explanations have been proposed to account for the emergence of the ER- breast cancer phenotype from tamoxifen treated ER+ breast cancers. ER point mutations are too infrequent to account for the high frequency of ER- breast cancers [20]. ER promoter hypermethylation is seen in some ER- cell lines and, although this does not appear to account for the ER- phenotype in most primary tumors, it should not be discounted as a possible cause of treatment-associated changes in ER phenotype. As it has been shown that ER- primary breast cancer expresses ER mRNA [21-23], it is possible that ER- tumors could arise through accelerated ER proteolysis. An hypothesis for the transformation of ER+ to ER- tumors during cancer progression may be that tamoxifen selects for the outgrowth of a neoplastic or pre-malignant breast cell subpopulation in which tamoxifen acts as a partial agonist to stimulate ER proteolysis.

Tamoxifen resistance has also been shown to emerge from a subpopulation in which epidermal growth factor receptor (EGFR) and/or Her2 mitogenic signaling is hyperactived. Transfection and activation of these receptors has been shown to confer antiestrogen resistance in breast cancer lines and xenografts [10,24,25]. Moreover, EGFR over-expression and Her2 amplification are both correlated with ER negativity in human breast cancers *in vivo *[26-29]. Data from the Slingerland lab suggest that both EGFR and Her2 signaling can cooperate with estrogen to activate ER proteolysis (I Chu, A Alkarain, J Sun, manuscript submitted). Thus, tamoxifen in the prevention setting may select for the outgrowth of EGFR or Her2 activated cancer cells that ultimately become ER- cancers. Experimental and clinical evidence support a model where tamoxifen can act as an agonist in a small fraction of cancers. Osborne and colleagues [30,31] have demonstrated that exposure to tamoxifen may accelerate the growth of tumors that are AIB1+. Jones and colleagues have shown that tamoxifen can be an agonist for the outgrowth of ER- hyperplasias and cancers in the MMTV-Cre *Brca1 *conditional exon 11 deletion model. An analogous situation could exist for precancerous cells [30,31].

In summary, the available data makes it difficult to exclude our hypothesis. Swain and colleagues [5] show that the ER status of primary tumors predicts the ER status of contralateral cancers that develop; however, women exposed to tamoxifen are less likely to develop ER+ tumors. The studies by Osborne *et al*. [30,31] suggest that the status of the first tumor does not predict the status of the second tumor, and that tamoxifen reduces the risk of a second tumor arising. Although these numbers are small, they suggest that ER- tumors arise preferentially because ER+ tumors are preferentially suppressed. Overview data suggest that exposure to tamoxifen has a lesser effect on the emergence of ER- tumors [1]. However, prolonged exposure to tamoxifen in the setting of early cancer increases the risk of cancers arising, although the ER status of those tumors is not known nor discoverable (J Costantino, personal communication). None of these observations preclude the possibility that some ER- tumors are prevented and that some ER- tumors may be stimulated by exposure to tamoxifen. This paper was designed to raise awareness among scientists and clinician researchers that there is another hypothesis that may explain the observed data, and to encourage them to be alert for and to explore observations of tamoxifen induced ER- cancers.

## Conclusion

We have shown that the BCPT and MORE data support the possibility that tamoxifen may exert selection pressure on some cells for the emergence of ER- cancers with activation of mitogenic signaling pathways. It is possible that these results are a mathematical coincidence, or an artifact of the data. If, however, the proposed hypothesis were true, the clinical implication is that while most women at high risk benefit from tamoxifen prophylaxis, there is a subset of women who would not benefit and might have ER- tumor transformation accelerated by prophylactic treatment with tamoxifen. These concepts provide stimulus for future investigations to identify the tumor cell phenotypes that differ in their susceptibility to preventive agents like tamoxifen. There may be subpopulations within normal or dysplastic but premalignant breast epithelium for which tamoxifen alone is insufficient prevention. It may be appropriate to limit tamoxifen therapy to the prevention of the most susceptible lesions, such as atypical ductal hyperplasia in which tamoxifen can reduce the risk of subsequent invasive disease by 80% [4]. We need to identify how carcinogens influence discrete cell subpopulations in the normal and atypical breast epithelium and to identify the susceptible cell types in order to tailor prevention efforts accordingly. Tamoxifen is likely to remain an important chemopreventive agent, particularly in the premenopausal setting. Thus, this hypothesis may help us to consider other combinations of agents for prevention, such as combinations of tamoxifen with small molecule inhibitors that target the EGFR family or novel receptor tyrosine kinases. More importantly, it should encourage the design of prevention interventions in a setting where we can follow biomarkers and prospectively test the hypothesis presented in this paper.

## Abbreviations

BCPT = Breast Cancer Prevention Trial; EGFR = epidermal growth factor receptor; ER = estrogen receptor; ER+ = estrogen receptor positive; ER- = estrogen receptor negative; MORE = Multiple Outcomes of Raloxifene; NSABP = National Surgical Adjuvant Breast and Bowel Project.

## Competing interests

The authors declare that they have no competing interests.

## Authors' contributions

LE conceived the study with the help of EO,. EO and LE designed the methods. EO ran the analyses. JS and MD contributed to the background and discussion, and provided insight about the results. All authors contributed to the writing of the manuscript and have approved the final manuscript.

## Figures and Tables

**Table 1:** Exposure to tamoxifen appears to affect the conditional likelihood of the estrogen receptor status of subsequent contralateral primary breast cancers

ER status of tumors		Tamoxifen treatment	
First cancer	Second cancer	No	Yes

ER-	ER-	19 (70%)	7 (78%)
	ER+	8 (30%)	2 (22%)
ER+	ER-	4 (11%)	17 (44%)
	ER+	31 (89%)	22 (56%)

Data were taken from the National Surgical Adjuvant Breast and Bowel Project trials B-18, B-22, and B-25 [5]. ER, estrogen receptor.

**Table 2:** Chemoprevention appears to prevent ER+ cancers, but has no effect on ER- cancers

ER status	Number of cancers^a^			
	Data from BCPT		Data from MORE	

	Placebo (175 breast cancers developed)	Tamoxifen (89 breast cancers developed)	Placebo (39 breast cancers developed)	Raloxifene (22 breast cancers developed)

ER-	31	38	4	9
ER+	130	41	31	10

^a^Because the estrogen receptor (ER) status was not know for all cancers, the numbers do not add up to the totals. Data were taken from [4,6]. BCPT, Breast Cancer Prevention Trial; MORE, Multiple Outcomes of Raloxifene.

**Table 3:** Calculation of Z using prevention trial data

Risk reduction	Z^a^	
	BCPT	MORE

50%	23.5%	26.6%
60%	20.1%	22.7%
70%	16.2%	18.2%
80%	11.6%	13.1%
90%	6.3%	7.1%

^a^Z is the rate of transformation from ER+ to ER- tumors. BCPT, Breast Cancer Prevention Trial; MORE, Multiple Outcomes of Raloxifene.
